# Effectiveness of Exercise and Local Steroid Injections for the Thoracolumbar Junction Syndrome (The Maigne’s Syndrome) Treatment

**DOI:** 10.2174/1874325001711010467

**Published:** 2017-05-31

**Authors:** Kerem Alptekin, Nurettin Irem Örnek, Tuğba Aydın, Mirsad Alkan, Mehmet Toprak, Leyla A. Balcı, Jülide Öncü Alptekin

**Affiliations:** Bahcesehir University, Health Sciences Faculty Department of Physiotherapy and Rehabilitation, Sahrayı Cedid Mahalesi Batman Sokak, Yenisahra/Kadıköy Istanbul, Turkey

**Keywords:** Maigne’s Syndrome, Corticosteroid injection, Exercise therapy, Back pain, Local anesthetic injection, Differential diagnosis

## Abstract

**Purpose:**

Patients diagnosed as thoracolumbar junction syndrome were divided into 3 treatment groups and the results of each modality were compared.

**Materials and Method:**

30 Patients were included in the study with the definitive diagnosis of Maigne’s Syndrome. The first group received exercise therapy, the second group was treated with local steroid injections and the third group was the combination therapy group of both injection and exercise.

**Findings:**

30 Patients were divided into 3 groups. Each group had 10 patients. The average age of the groups was detected to be 23.43 ± 3.75. A flattening was detected in 4 patients of the first group (40%), 6 patients of the second group (60%) and 4 patients of the third group (40%) during the lumbar lordosis. While the average difference of the VAS values was (2.80) as the lowest for the injection group before and after treatment at rest, the highest value (3.30) was observed in the combined treatment group. The results shown on the Oswestry scale of the first month difference (16.10), and the third month difference (22.40) were statistically better than the other groups in the combined treatment group.

**Results:**

As a result of this study, while in all three treatment groups in the Oswestry scale, VAS scores at rest or at movement during the regular controls before and after the treatment showed statistically significant difference; the best results were obtained in the group administered to the combined injection and exercise therapy.

## INTRODUCTION

The Maigne’s Syndrome also known as the thoracolumbar junction syndrome (TLS) caused by thoracolumbar lateral nerve branch was first described by Maigne [1]. TLS clinical findings showed that the affected movement segment includes sensitivity with palpation at the (T12 and L3) point with the pain radiating throughout segmental nerve distribution originated by the thoracolumbar (dorsal or ventral ramie sections). The pain radiation and clinical results show that the distribution of the spinal nerve root T12 and L1; while the posterior branch of L1 innervates the lower lumbar subcutaneous tissue, the anterior branch innervates the groin region, and the lateral cutaneous branch innervates the lateral hip side [2] Fig. (**1**).

Maigne [3] characterized thoracolumbar junction syndrome by specific physical findings. The clinical diagnosis may be reaffirmed in one or more thoracolumbar region (between T11-L3 vertebrae) by the tenderness on palpation test; comparing the sensitivity difference on the iliac crest (cluneal nerve), the inguinal canal (inguinal nerve) or the greater trochanter (lateral perforator nerve) and by rolling and tightening the skin on the normal side. Skin rolling test or German origin Kiblerfalte test procedure is: The patient lies prone with arms relaxed alongside the trunk. The examiner raises a fold of skin between thumb and forefinger and “rolls” it along the trunk or, on the extremities, perpendicular to the course of the dermatomes. The examiner compares both sides and states where the skin is rolled more heavily [4].

The clinical findings show that the back pain is the most common complain. Due to interference between the back pain and the waist pathologies, the hip pain and the hip pathologies, the pubic pain and the hip adductor pathology, the TLS may be overlooked. Besides, since the lower abdomen is affected while causing a pseudo visceral pain, the pain could be imitated with induced pains causing gynecological, gastroenterological and urological pains. A definitive diagnosis may be provided by the local anesthetic injection in the thoracolumbar region with cessation of the current pain after injection. The strength and stability should be improved by muscle balance and stabilization exercises. We aimed in our study to search the effectiveness of the exercise, and local steroid injection treatment as a TLS treatment modality [5].

## THORACOLUMBAR JUNCTION SYNDROME ETIOLOGY

The thoracic facet joint irritation is a common cause of pain at the lower lumbar and lumbosacral region [6].

The 12^th^ thoracic vertebra being a transition segment is located between the lumbar facets at the sagittal plane with the thoracic facet in the coronal plane. The 12^th^ thoracic vertebra continues to be the focus of the transitional stress during the spinal movements. This stress results as traumatic lesions at the T9-L2 facet joints and may cause unilateral radiating pain in the lower lumbar and upper gluteal region [7].

While standing on the floor of the lower extremities, the professional activities in the form of rotating the spine that can lead to repetitive loading of the spine (such as hairdressing conglutinating *etc*.) and sports activities (such as hockey *etc*), creating stress on the thoracolumbar junction can lead to injury. Also, it has been reported that bending the head forward and back with hip flexion during sports activities of the thoracolumbar spine will be beneficial [8].

When the thoracolumbar region is overloaded, the adjacent lamina can be affected by the inferior articular process and as a result, the contralateral posterior joint capsule will be stretched and pain can occur [9].

The pain dissemination and clinical results are related to the T12 and L1 anatomical distribution of the spinal nerve root; while the posterior branch of the upper gluteal innervates lower lumbar subcutaneous tissues; it innervates the anterior region of the lower abdomen and the crotch region, and the lateral cutaneous innervates the outer side of the hip [2].

The musculoskeletal entities to consider for the cause of back pain include: compromise of the anterior spinal nerves or posterior primary rami of thoracolumbar origin, pathology of the thoracolumbar zygapophyseal joints and/or their joint capsules, thoracolumbar disc disruption, congenital malformations, degenerative processes and fibromyalgia. It is important in patients subjected to significant trauma to rule out vertebral fracture [10] (*i.e*. Chance, burst or compression type) and/or spinal instability.

In the differential diagnosis, musculoskeletal diseases need to be paid attention which cause the back pain and are as follows: thoracolumbar zygapophyseal joint and/or the capsule joint, thoracic disk degradation, congenital malformations, degenerative processes and the pathology of the anterior spinal nerves or a thoracolumbar reconciliation originated from a posterior primary ramie, and the fibromyalgia. It is important to examine patients who may have been exposed to a major trauma in order to control this vertebral fracture [11] (*i.e*. chance, explosion or compression type) and / or the spinal instability.

Patients found in the clinical application complain about the deteriorating hip pain and back pain from time to time while walking. In such case, the diagnosis of the disease together with the difficulties, definition of the pain originated from the thoracolumbar region is possible in 5 steps;

1- Iliac crest point finding
2- Skin rounding test
3- The findings of the specific level of involvement in the thoracolumbar region
4- Facet joints palpation
5- Local anesthetic injection [12];

İliac crest is easily found by palpating the lateral margin of iliac bone. On the other hand, spinous process of L4 is palpated and followed lateral till the crest is marked [13]. The specific findings of thoracolumbar region are the pain pathways radiating to upper gluteal, lower lumbar or inguinal region. In addition, the TLS is indicated in case of flexion in lateral worsening symptoms with back extension in patients’ lateral [4]. Facet joint palpation is not an easy technique, because they are located 4-6 cm below the skin. For example, L4-L5 facet joint is located by first palpating L4 spinous process and following 2-3 cm laterally on right and left side .

## TREATMENT MODALITIES USED IN THORACOLUMBAR JUNCTION SYNDROME

The manual therapy applications, and the exercises and injection applications are most often preferred treatment methods during the treatment of disease.

The corticosteroid injection in the treatment which has widespread use shows positive effects of early and late periods at the Facet joint pathology. The edema, the fibrin formation, the capillary dilatation and the leukocyte aggregation and phagocytosis occur from the effects of the Corticosteroids early inflammation; on the other hand, preventing the late effects collagen formation and scatrisation by capillaries and fibroblast proliferation plays an important role in controlling the inflammation. The intermediate materials involved within the C fibers and nociception can be suppressed by corticosteroid injection while being effective in controlling the inflammation. Due to this aspect, the suppression of the corticosteroids for acute and chronic pain in the suppression of pain offers a segmental and clear solution while being an important tool [11]. During the first stage, the corticosteroids showing equal effect on the acute and chronic pain have the specialty of decreasing the rates to 34% in the acute pain and 12% in the chronic pain the at the 6-month follow-up process [4]. On the other hand, for some patients who were given the acting corticosteroid injection and were provided with the facet where an involvement is noticed, it has been reported to act as a substitute manipulation in Maigne’s syndrome [12].

The 4 steps functional restoration program for the TLS commonly seen in athletes can be defined as follows;

1- Elimination of pain and inflammation;
Cold and electrical stimulation
Nonsteroidal anti-inflammatory drugs
Posture training
Myofascial therapy
2- Regaining the range of motion
Manual medical applications
Providing flexibility and muscle force balance
Dissociative movement therapy (beginning level)
Simple stabilization
Walking
3- Gaining muscle strength and muscle strength balance
Intermediate and advanced stabilization
Proprioceptive exercises
Dissociative movement therapy (intermediate and advanced)
Plyometric exercises
Resistance exercises / free weight exercises
4- Return to work/sport
Specific activities at work/sport [4]

The exercises provided in Maigne’s syndrome are formed by applying 4 different modifications of standard pelvic tilt exercises and such modifications are provided step by step and recommended to the patients, respectively [14]. The patient cannot pass to the next step without completing the previous step of doing the exercise smoothly, securely and distinctly considering the pain (VAS from 0 to10 and 5 and above identifies considerable pain). The manner in which the exercise is done is as follows: for the pelvic tilt exercises 10 times and for each muscle group, it needs 25 seconds of execution movement; also for the hip lifting exercises, it is 10 times but the time is recommended to be 10 seconds. The most important thing is that the exercises should be done in the morning and, if possible, after waking up before getting out of bed. However, the exercises are not suitable for all the patients, especially those of neurodegenerative diseases originated from coordination disorders depending on the extent of making counter-indications for coordination disorder. 4 different modifications of the pelvic tilt exercises given to the patients are completed in 6 steps, but during the study, those exercises have been applied to patients for the first three steps only. After that, the hip lifting exercises are given to patients.

- Pelvic tilt exercises
  1. Step - simple pelvic tilt exercises
  2. Step - the addition of the pelvic muscles to the simple pelvic tilt exercise
  3. Step - the addition of the back extension exercises to the first two steps exercise
  4. Step - the operation of the deep neck flexors and extensors
  5. Step - the addition of deep neck muscles plans to the first three steps exercise
  6. Step - positioning the hands to the back when you exercise

B. Hip-bearing exercise

## MATERIALS AND METHOD

The patients who applied for the Physical Medicine and Rehabilitation clinic and who had complains regarding their waists, hips, flanks or in their groins, differential diagnoses were examined on them which resulted in low back pain and lumbar spinal magnetic resonance visualization. By excluding any other diseases which did not have any pathology or had only flattening of the lumbar lordosis, the physical examination and the thoracolumbar junction were performed with local anesthetic injection showing the definitive diagnosis of Maigne’s Syndrome in patients with ages ranging between 19-33 years; among them, 37 patients were evaluated for their suitability to the study and a total of 30 patients (1 female, 29 male) were included in the study. Positive injection response to local anesthestics and exclusion of other lumbar diseases with MRI were the key point of the patient selection. 7 Patients were excluded from the study. The reasons for exclusion are mentioned in the next paragraph.

According to the stories of the 6 month short-term patients complaining about the pain in the waist, hips and groin or the outer side region and according to the lumbar MRI results, the patients who were normal or having flattening in the lumbar lordosis only were excluded. The patients who had precisely TLS diagnosed and whose ages ranged between 19-33 years were examined by a local anesthetic injection and were provided with the thoracolumbar junction, being included in the study. The patients who had complains regarding the back, waist and the outer side of the hip and groin pain for longer than 6 months, 33 years and older patients who had a body mass index equaling to (BMI) 30 kg/m^2^ and above 18.5 kg/m^2^ or less were excluded from the study. Also, 2 of the 4 patients who still had unresolved complaints in the local anesthetic injection induced in the thoracolumbar region had chronic disease and 1 of the patients was excluded since he did not follow the exercise program.

**Fig. (2):**
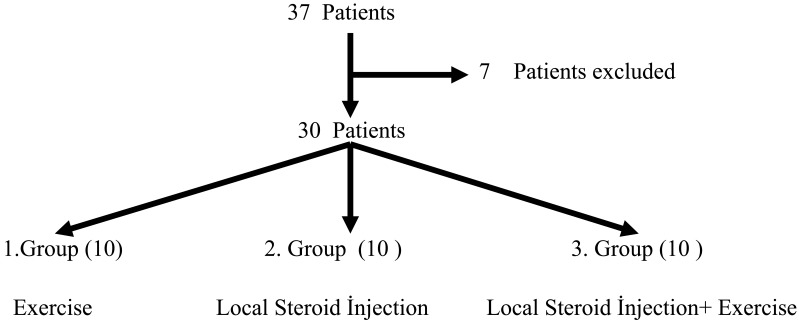
Study design and patient allocation.

The treatment given to the first group of patients of the 3 group patients was just exercise therapies. Only local steroid treatment was applied to the patients in the second group from the thoracolumbar junction region and exercise therapy was not provided. The third group patients were provided by the local steroid treatment and exercise therapy. At the beginning of the study, 37 patients were evaluated and 30 patients were suitable for the study. They were randomly allocated to the three groups and each group consisted of 10 patients (Fig. **2**).

The recommended exercises for patients include: the 1^st^ step involving simple pelvic tilt, the 2^nd^ step being simple pelvic tilt with the addition of pelvic muscle exercises and 3^rd^ step of exercise including the first two steps with addition of the back extension.

Since the facet joint injection was applied to the patients during our study, the local steroid injection was induced to the patients receiving the injection therapy in the thoracolumbar junction region; yet the manual therapy applications have not been applied. Besides exercise therapy, no manual therapy methods were used.

The patients in the exercise group therapy conducted the exercises during their control visits and they were followed carefully regarding the exercises they could not do. The patients who were given the exercise programs were followed up regarding their frequency and regularity of doing the exercises. The lumbar range of motion (ROM), the visual analogue scale at rest and during movement (VAS) values, the Beck Depression Scale, Oswestry scales and the SF-36 results values of the patients were evaluated before and after injection, during the 1^st^ week, the 1^st^ month and 3^rd^ month control visits.

## FINDINGS

**The Statistical Methods:** The highest values are used in the descriptive statistics of the data average, standard deviation and the lowest median. The distribution of the variable is measured by the Kolmogorov-Smirnov test. The ANOVA (Tukey test), Kruskal-Wallis and Mann-Whitney U test were used during the Quantitative data analysis. The SPSS 22.0 program was used during the analysis.

No significant difference was found between Groups I, II and III regarding patients’ ages and BMI (p ˃ 0.05) (Table **1**).

The lumbar flexion value of the Group I, Group II and Group III shows significant differences (p ˃ 0.05) in the pretreatment and the post-injection during the 1^st^ week and the 4^th^ week. The lumbar flexion value of Group I in the 3^rd^ month shows higher value compared with the lumbar flexion value of Group II and Group III (p ˂ 0.05). The lumbar flexion value of Group II and Group III in the 3^rd^ month shows significant differences (p ˃ 0.05) (Table **2**).

The lumbar extension value shows significant differences (p ˃ 0.05) during the post-injection of the Group I, Group II and Group III. The lumbar extension value Group I in the 1^st^week, 4^th^ week, 3^rd^ week shows a higher value from that in the Group II and Group III (p ˂ 0.05). The lumbar extension value in the Group II and Group III during the 1^st^ week, the 4^th^ week and the 3^rd^ month shows significant differences (p ˃ 0.05) (Table **2**)

The lumbar lateral flexion shows significant differences (p ˃ 0.05) during the post-injection of the Group I, Group II and Group III in the 1^st^ week. The lumbar lateral flexion in the Group I shows a higher value than that in the Group II and Group III during 4^th^ week and 3^rd^ week (p ˂ 0.05). The lumbar lateral flexion value in the Group II shows significant differences (p ˃ 0.05) than that in the Group III during the 4^th^ week and the 3^rd^ month (Table **2**).

The lumbar rotation value shows significant differences (p ˃ 0.05) during the pretreatment and the post-injection of the Group I, Group II and Group III. The lumbar rotation value in the Group I and Group II during the 1^st^ week, the 4^th^ week and the 3^rd^ month shows significant differences (p ˂ 0.05) compared with that in the Group III. The lumbar rotation value of the Group I during the 4^th^ week and the 3^rd^ month shows a higher difference (p ˂ 0.05) when compared with that in the Group II. The lumbar rotation value of the Group I and Group II during the 1^st^ week shows significant differences (p ˂ 0.05) (Table **2**).

The rest VAS value shows significant differences (p ˃ 0.05) during the pretreatment and the post-injection of the Group I, Group II and Group III. The rest VAS value in the Group I and Group II during the 1^st^ week shows significant differences (p ˂ 0.05) compared with that in the Group III. The rest VAS value of the Group I and Group II shows significant differences during the 1^st^ week. The rest VAS value of the Group I during the 4^th^ week and the 3^rd^ month shows significant differences (p ˂ 0.05) compared with that in the Group II and Group III. The rest VAS value of the Group II and Group III shows significant differences during the 4^th^ week and the 3^rd^ month (Table **3**).

The movement VAS value shows significant differences (p ˃ 0.05) during the pretreatment and the post-injection of the Group I, Group II and Group III. The movement VAS value of Group I and Group II during 1^st^ week shows significant differences (p ˂ 0.05) compared with that of the Group III. The movement VAS value of the Group I and Group II shows significant differences (p ˃ 0.05) during the 1^st^ week. The movement VAS value of the Group I during the 4^th^ week and the 3^rd^ month shows significant differences (p ˂ 0.05) compared with that of the Group II and Group III. The movement VAS value of the Group II and Group III shows significant differences (p ˂ 0.05) during the 4^th^ week and the 3^rd^ month (Table **3**).

The Beck depression score shows significant differences (p ˃ 0.05) during the pretreatment and the post-injection of the Group I, Group II and Group III (Table **3**)

The OSWESTRY score shows significant differences (p ˃ 0.05) during the pretreatment Group I, Group II and Group III. The OSWESTRY score of the Group I and Group II shows significant differences (p ˃ 0.05) during the 1^st^ month and the 3^rd^ month (Table **3**).

The SF-36 general health score of the Group I, Group II and Group III shows significant differences (p ˃ 0.05) during the pretreatment. The SF-36 general health score of the Group I and Group II shows significant differences (p ˃ 0.05) less than that in Group III during the 1^st^ month and the 3^rd^ month. The SF-36 general health score of the Group I shows significant differences (p ˃ 0.05) less than that in Group II during the 1^st^ month and the 3^rd^ month (Table **4**).

The SF-36 previous Health score of the Group I, Group II Group III in the pretreatment shows a significant difference (p ˃ 0.05). (Table **4**)

The SF-36 activity restriction score of the Group I, Group II and Group III in the pretreatment shows a significant difference (p ˃ 0.05). The SF-36 activity restriction score of the Group I and Group II shows a significant difference (p ˃ 0.05) less than that in the Group III during the 1^st^ month and the 3^rd^ month. The SF-36 activity restriction score of the Group I and Group II shows a significant difference (p ˃ 0.05) during the 1^st^ month. The SF-36 activity restriction score of the Group shows a significant difference (p ˃ 0.05) less than that in the Group II during the 3^rd^ month (Table **4**).

The SF-36 Activity problem score of the Group I, Group II and Group III in the pretreatment shows a significant difference (p ˃ 0.05). The SF-36 Activity problem score of the Group I and Group II during the 1^st^ month and the 3^rd^ month shows a significent difference (p ˃ 0.05) less than that in the Group III. The SF-36 Activity problem score of the Group I shows a significant difference (p ˃ 0.05) less than that in the Group II during the 1^st^ month and the 3^rd^ month (Table **4**).

The SF-36 emotional score of the Group I, Group II and Group III in the pretreatment shows a significant difference (p ˃ 0.05). The SF-36 emotional score of the Group I and Group II during the 1^st^ month and the 3^rd^ shows a significant difference (p ˂ 0.05) less than that in the Group III. The SF-36 emotional score of the Group I shows a significant difference (p ˃ 0.05) less than that in the Group II during the 1^st^ and the 3^rd^ month (Table **4**).

The SF-36 social relationship score of the Group I, Group II and Group III in the pretreatment shows a significant difference (p ˃ 0.05). The SF-36 social relationship score of the Group I and Group II in the pretreatment shows a significant difference (p ˃ 0.05) during the 1^st^ month. The SF-36 social relationship score of the Group I during the 3^rd^ month shows a significant difference (p ˂ 0.05) less than that in the Group II (Table **4**).

The SF-36 previous pain score of the Group I, Group II and Group III in the pretreatment shows a significant difference (p ˃ 0.05) during the 1^st^ month and the 3^rd^ month (Table **4**).

The SF-36 pain life Score of the Group I, Group II and Group III in the pretreatment shows a significant difference (p ˃ 0.05) during the 1^st^ month and the 3^rd^ month. (Table **4**)

The SF-36 Health Score of the Group I, Group II and Group III in the pretreatment shows a significant difference (p ˃ 0.05). The SF-36 Health Score of the Group I and Group II during the 1^st^ month and the 3^rd^ month shows a significant difference (p ˂ 0.05) less than that in the Group III. The SF-36 Health Score of the Group I and Group II shows a significant difference (p ˃ 0.05) during the 1^st^ month. The SF-36 Health Score of the Group I shows a significant difference (p ˃ 0.05) less than that in the Group II during the 3^rd^ month (Table **4**).

The 1 month and 3 months SF-36 previous sense score (p ˃ 0.05) has not shown any meaningful variation on the pretreatment of Group I, Group II and Group III (Table **4**).

## DISCUSSION

The most common levels T11-T12, T12-L1 which are usually considered as unilateral and sometimes as bilateral can be defined as the TLS involvement showing that according to the Maigne minor vertebral disorders, and according to chiropractic subluxation and osteopathy, they are somatic [12]. But if the TLS is a matter to be discussed by all the related methods, then the three perspectives would not be adequate for defining the disease. Although the examination of a certain area of the affected muscle is helpful in understanding it in a better way, and even though the compliance of the joint surfaces is corrupt, but there is not a disorder that can be defined in subluxation degree. Finally, although the somatic dysfunction could be seen within the context in which Chapmann was described but the disease is not just that.

The Corticosteroid injections common usage is one of the methods for controlling inflammation and pain at the early and late periods. The traumatic lesions of the thoracolumbar junction play an important role in the Maigne’s syndrome etiology. Therefore, many positive results are to be expected by the implementation corticosteroid injection in the traumatic lesions region at the Maigne’s syndrome. Despite that, it is seen that it is not an adequate treatment alone according to the decrease in the long-term success of corticosteroid and considering their side effects. This is because of the loss of spinal stabilization due to the muscular insufficiency which is considered as the main problem and due to other mechanical causes regarding the mechanical back and low back pain. The application results of corticosteroid injection are summed up as the pain and inflammation reduction which enhances the effectiveness of exercise through strengthening of the muscles and the thoracolumbar region, so they have been considered as satisfactory treatment effects for corticosteroids [15].

## CONCLUSION

In our study, the exercises given to the patients can primarily affect the facet joints found in the upper lumbar region with the multifidus, the quadratus lumbar, and the pelvic floor muscles. By judging from a much wider angle, the deterioration of sexual function in young adults regarding the weakness of these muscles up to the urinary incontinence development occurring in much older individuals has shown that the patients had various negative effects in the patients applying to the mentioned muscle clinics with complaints of back pain. In this aspect, the exercise mentioned within the study could be beneficial not just for the Maigne’s syndrome patients only but also for a wider population. On the other hand, these exercises provide the advantage of doing other exercises at the same time in order to face the growing differences in muscle length, the elimination of muscle strength imbalance and the development of proprioception.

During the development of the Maigne’s syndrome, the facet or muscles joints are effective to get rid of the mechanical stress when doing the hip flexions with the head extension simultaneously. There are some programs which require some exercise positions in some functional rehabilitation tools, which enable to prevent all kind of loss of balance during the exercises. Therefore, applying the functional rehabilitation equipment hosted by the Software-based systems or the mechanical systems hosted by the balance board assembly on these patients may lead to worsening of the symptoms.

As a result of bone diseases such as the osteogenesis imperfecta and osteoporosis which have been reported in the literature, the developing T11 has been taken into consideration in cases such as the thoracolumbar junction syndrome due to the compression fractures. Regarding the risk of the manipulative treatment applications in the developing thoracolumbar junction syndrome related to the compression fractures, it has been reported that the manipulative therapy did not show adequate effect when used by itself in the healthy cases [10].

On the other hand, the presence of muscular dystrophy results, the possible muscular weakness results and the thoracolumbar junction problems may occur again depending on the same disease and the changes in bone density. It can be seen that the muscular dystrophy should be taken into consideration before the manipulation or exercise therapies in the treatment.

A small number of case reports have been shown that it is successful in relation to the corticosteroid injection practices in the thoracolumbar junction syndrome in literature scan. Although insufficient sample group during the studies is required to be less than the needed studies number, the study results are such as to overlap the problems reported in the literature. However, new studies must be conducted on different populations regarding this topic.

## Figures and Tables

**Fig. (1):**
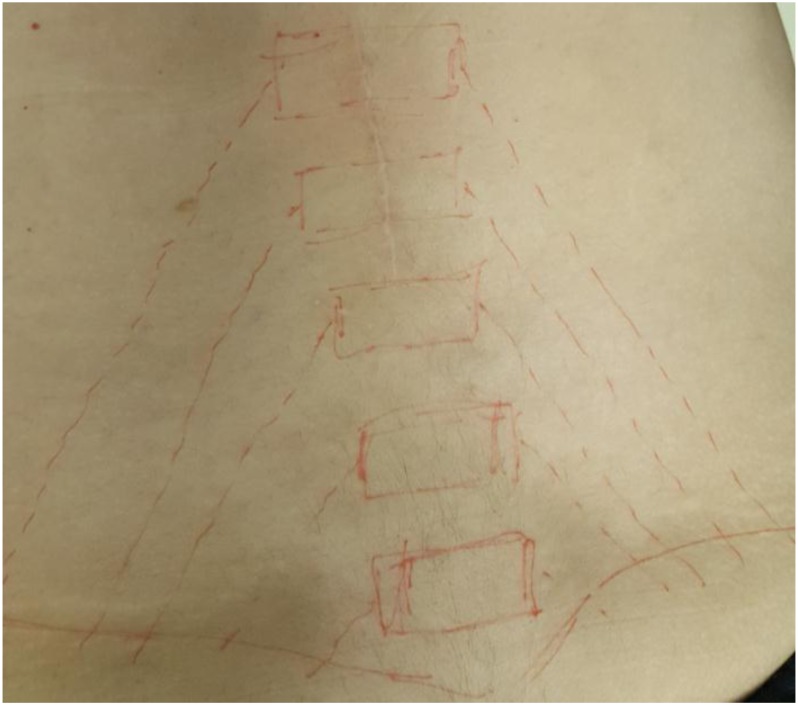
Topographic presentation of dorsal rami.

**Table 1:** Baseline values of age and BMI of 3 groups.

	**Group I**		**Group II**		**Group III**		**p**
**Age**	**Avg. ± s.s.**	**Median**	**Avg. ± s.s.**	**Median**	**Avg. ± s.s.**	**Median**	
**BMI**	23.4 ± 3.0	22.5	23.2 ± 4.3	211.0	23.1 ± 3.4	22.0	0.823
	23.5 ± 2.5	23.2	23.4 ± 2.5	23.1	23.5 ± 3.6	22.5	0.992
**ANOVA / Kruskal-wallis**							

**Table 2:** Lumbar range of motion pretreatment and posttreatment values.

	**Group I**		**Group II**		**Group III**		p
	**Avg. ± s.s.**	**Median**	**Avg. ± s.s.**	**Median**	**Avg. ± s.s.**	**Median**	
**Lumber FLX**							
Pretreatment	15.0 ± 7.1	20.0	15.6 ± 12.4	10.0	16.7 ± 19.4	10.0	0.0962
Post-Injection	5.0 ± 5.3	5.0	7.2 ± 9.7	0.0	9.4 ± 11.3	5.0	0.786
1^st^ week	11.0 ± 5.7	10.0	6.7 ± 10.0	0.0	8.9 ± 11.7	0.0	0.405
4^th^ week	12.0 ± 6.3	10.0	6.7 ± 10.0	0.0	6.1 ±8.6	0.0	0.168
3^rd^ month	12.0 ± 6.3	10.0	4.4 ± 7.3	0.0	3.3 ± 5.2	0.0	***0.014***
**Lumber EXT**							
Pretreatment	35.0 ± 14.3	35.0	31.1 ± 12.7	30.0	34.4 ± 23.5	30.0	0.796
Post-Injection	23.0 ± 11.6	20.0	23.3 ± 12.2	20.0	20.0 ± 13.2	20.0	0.937
1^st^ week	26.0 ± 10.7	25.0	16.7 ± 12.2	10.0	10.0 ± 113.2	0.0	***0.035***
4^th^ week	25.0 ± 9.7	30.0	10.0 ± 10.0	10.0	5.6 ± 8.8	0.0	***0.002***
3^rd^ month	25.0 ± 9.7	30.0	7.8 ± 6.7	10.0	3.8 ± 5.2	0.0	***0.000***
**Lumber Lat FLX**							
Pretreatment	25.0 ± 5.3	25.0	32.2 ± 9.7	30.0	31.11 ± 20.9	30.0	0.127
Post-Injection	16.0 ± 7.0	15.0	20.0 ± 7.1	20.0	18.9 ± 18.3	20.0	0.509
1^st^ week	19.0 ± 7.4	20.0	16.7 ± 8.7	20.0	7.8 ± 10.9	0.0	0.051
4^th^ week	19.0 ± 7.4	20.0	11.11 ± 7.8	10.0	4.4 ± 10.1	0.0	***0.005***
3^rd^ month	17.0 ± 6.7	20.0	10.0 ± 7.1	10.0	2.2 ± 6.7	0.0	***0.001***
**Lumber Rot**							
Pretreatment	26.0 ± 7.0	25.0	28.9 ± 9.3	30.0	24.4 ± 15.9	20.0	0.352
Post-Injection	14.0 ± 7.0	10.0	18.9 ± 6.0	20.0	13.3 ± 10.0	10.0	0.206
1^st^ week	18.0 ± 7.9	20.0	15.6 ± 8.8	20.0	5.6 ± 5.3	0.0	***0.006***
4^th^ week	20.0 ± 8.2	20.0	10.0 ± 7.1	10.0	1.1 ± 3.3	0.0	***0.000***
3^rd^ month	18.0 ± 7.9	20.0	8.9 ± 6.0	10.0	1.1 ± 3.3	0.0	***0.000***
**Kruskal-wallis (Mann-whitney u test)**							

**Table 3:** Pretreatment and posttreatment VAS, Beck Depression, Oswestry scores.

	**Group I**		**Group II**		**Group III**		**p**
	**Avg. ± s.s.**	**Median**	**Avg. ± s.s.**	**Median**	**Avg. ± s.s.**	**Median**	
**Rest VAS**							
Pretreatment	5.2 ±	5.0	5.8 ± 1.3	6.0	5.1 ± 1.2	5.0	0.308
Post-Injection	2.0 ±	2.0	3.0 ± 1.6	4.0	1.3 ± 1.1	1.0	0.056
1^st^ week	3.8 ±	4.0	3.2 ± 1.3	4.0	1.8 ± 1.3	1.0	***0.006***
4^th^ week	3.8 ±	4.0	1.9 ± 1.8	1.0	1.0 ± 0.9	1.0	***0.001***
3^rd^ month	4.1 ±	4.0	1.7 ± 1.7	1.0	0.3 ± 0.5	0.0	***0.000***
**Movement VAS**							
Pretreatment	7.2 ± 1.0	7.0	7.4 ± 1.0	8.0	7.1 ± 0.9	7.0	0.539
Post-Injection	3.5 ± 1.6	4.0	4.4 ± 2.2	5.0	2.6 ± 1.3	2.0	0.069
1^st^ week	5.8 ± 0.9	6.0	4.7 ± 1.7	5.0	3.1 ± 1.6	3.0	***0.003***
4^th^ week	5.7 ± 0.8	5.0	2.9 ± 2.2	2.0	1.7 ± 1.5	2.0	***0.000***
3^rd^ month	6.0 ± 1.1	6.0	2.8 ± 1.9	2.0	0.9 ± 1.1	1.0	***0.000***
**Beck Depresion**							
Pretreatment	27.6 ± 8.2	30.5	37.2 ± 7.5	35.0	29.9 ± 12.3	28.0	0.101
1^st^ month	26.4 ± 7.2	29.0	34.7 ± 7.2	35.0	25.9 ± 12.6	26.0	0.133
3^rd^ month	26.1 ± 5.0	27.0	30.0 ± 6.2	30.0	23.2 ± 12.2	22.0	***0.367***
**OSWESTRY Score**							
Pretreatment	37.8 ±3.5	39.0	38.0 ± 5.7	38.0	40.6± 3.5	39.0	0.446
1^st^ month	35.1 ± 3.8	35.5	35.1 ± 5.1	35.0	24.6 ± 5.8	24.0	***0.002***
3^rd^ month	34.9 ± 4.7	36.5	31.1 ± 4.4	32ç0	18.1 ± 4.2	19.0	***0.000***
**Kruskal-wallis (Mann-whitney u test)**							

**Table 4:** SF-36 scores before and after treatment.

**SF-36**	**Group I**		**Group II**		**Group III**		**p**
	**Avg. ± s.s.**	**Median**	**Avg. ± s.s.**	**Median**	**Avg. ± s.s.**	**Median**	
**General Health Score**							
Befor Treatment	31.0 ± 10.5	30.0	32.8 ± 17.5	30.0	27.8 ± 10.8	25.0	0.697
1^st^ Month	42.5 ± 11.6	45.0	53.9 ± 18.0	50.0	65.0 ± 13.5	65.0	**0.004**
2^nd^ Month	48.0 ± 9.5	47.5	69.4 ± 13.6	70.0	82.0 ± 11.2	80.0	**0.000**
**Previous Health**							
Before Treatment	12.5 ± 17.7	0.0	33.3 ± 33.1	25.0	8.3 ± 12.5	0.00.0	0.090
1^st^ Month	32.5 ± 29.0	25.0	33.3 ± 33.1	25.0	58.3 ± 21.7	50.0	0.074
2^nd^ Month	50.0 ± 40.8	37.5	47.2 ± 34.1	25.0	80.6 ± 24.3	75.0	0.107
**Activity Restrictions**							
Before Treatment	16.2 ± 10.6	22.0	15.1 ± 16.6	10.0	25.3 ± 17.3	311.0	0.285
1^st^ Month	27.7 ± 10.3	22.0	30.6 ± 12.4	31.0	62.4 ± 22.6	52.0	**0.000**
2^nd^ Month	26.3 ± 12.8	22.0	45.0 ± 15.9	41.0	79.6 ± 12.8	74.0	**0.000**
**Activity Problems**							
Before Treatment	29.7 ± 18.4	17.5	27.2 ± 17.7	20.0	29.7 ± 22.0	35.0	0.973
1^st^ Month	30.5 ± 15.0	20.0	23.3 ± 9.4	25.0	45.4 ± 18.3	47.0	**0.027**
2^nd^ Month	28.7 ± 15.4	20.0	32.0 ± 14.0	37.0	48.4 ±16.8	52.0	**0.000**
**Emotional**							
Before Treatment	29.0 ± 22.3	20.0	31.7 ± 22.2	35.0	40.0 ± 16.8	50.0	0.422
1^st^ Month	28.5 ± 18.3	20.0	22.8 ± 23.9	10.0	48.3 ± 16.8	50.0	**0.027**
2^nd^ Month	28.0 ± 12.7	25.0	27.8 ± 24.1	20.0	50.0 ± 15.0	50.0	**0.031**
**Social relations**							
Before Treatment	25.0 ± 21.2	18.8	38.9 ± 25.3	37.5	34.7 ± 15.0	37.5	0.231
1^st^ Month	33.8 ± 19.6	25.0	33.3 ± 18.8	37.5	59.7 ± 16.3	50.0	**0.026**
2^nd^ Month	33.8 ± 11.9	31.3	44.4 ± 11.0	50.0	66.7 ± 21.7	62.5	**0.031**
**Pain**							
Before Treatment	33.3 ± 22.2	33.3	33.3 ± 23.6	33.3	37.0 ± 15.0	33.3	0.993
1^st^ Month	30.0 ± 18.9	33.3	37.0 ± 23.6	33.3	51.9 ± 29.4	66.7	0.221
2^nd^ Month	33.3 ± 31.4	33.3	37.0 ± 30.9	33.3	51.9 ± 29.4	66.7	0.302
**Pain Life**							
Before Treatment	43.6 ± 25.5	36.0	39.6 ± 26.4	48.0	47.6 ± 18.9	48.0	0.698
1^st^ Month	44.0 ± 24.7	36.0	34.7 ± 21.4	32.0	47.6 ± 18.3	48.0	0.408
2^nd^ Month	40.8 ± 19.4	38.0	34.7 ± 21.4	32.0	49.3 ± 17.5	52.0	0.269
**Health**							
Before Treatment	26.11 ± 3.6	25.7	28.6 ± 6.7	26.5	25.7 ± 6.7	26.0	0.586
1^st^ Month	32.0 ± 5.3	31.0	34.4 ± 6.0	33.1	43.9 ± 7.4	41.4	**0.005**
2^nd^ Month	34.7 ± 7.8	34.0	41.8 ± 5.4	41.2	51.9 ± 4.7	53.0	**0.000**
**Feeling**							
Before Treatment	35.3 ± 11.7	31.4	35.3 ± 9.8	37.5	39.3 ± 10.1	37.9	0.576
1^st^ Month	33.6 ± 11.8	28.2	30.1 ± 98.4	29.8	37.4 ± 9.0	39.6	0.291
2^nd^ Month	32.0 ± 9.3	28.3	29.2 ± 8.1	28.3	36.11 ± 8.1	40.6	0.198
**Kruskal-Wallis (Mann-whitney u test)**							

## LIST OF ABBREVIATIONS

BMI: = Body Mass Index
MRI: = Magnetic Resonance Imaging
ROM: = Range of Motion
SF-36: = Short Form-36
TLS: = Thoracoloumbar Junction Syndrome
VAS: = Visual Analogue Scale
